# A Secure Multi-Tier Mobile Edge Computing Model for Data Processing Offloading Based on Degree of Trust

**DOI:** 10.3390/s18103211

**Published:** 2018-09-23

**Authors:** Francisco José Mora-Gimeno, Higinio Mora-Mora, Diego Marcos-Jorquera, Bruno Volckaert

**Affiliations:** 1 Department of Computer Technology and Computation, University of Alicante, 03690 Alicante, Spain; hmora@dtic.ua.es (H.M.-M.); dmarcos@dtic.ua.es (D.M.-J.); 2 Department of Information Technology, Ghent University, 9052 Ghent, Belgium; bruno.volckaert@intec.ugent.be

**Keywords:** mobile edge computing, security models, data processing offloading, multi-tier architectures

## Abstract

Current mobile devices need to run applications with high computational demands and critical response times. The mobile edge computing (MEC) paradigm was developed to improve the performance of these devices. This new computation architecture allows for the mobile devices to execute applications on fog nodes at the network edge; this process is called data processing offloading. This article presents a security model for the externalization of application execution in multi-tier MEC environments. The principal novelty of this study is that the model is able to modify the required security level in each tier of the distributed architecture as a function of the degree of trust associated with that tier. The basic idea is that a higher degree of trust requires a lower level of security, and vice versa. A formal framework is introduced that represents the general environment of application execution in distributed MEC architectures. An architecture is proposed that allows for deployment of the model in production environments and is implemented for evaluation purposes. The results show that the security model can be applied in multi-tier MEC architectures and that the model produces a minimal overhead, especially for computationally intensive applications.

## 1. Introduction

In recent years, increasing attention has been paid to the use of the mobile edge computing (MEC) paradigm. The expansion of the Internet of Things and the deployment of 5G communications will increase the adoption of MEC systems in society. Despite the constant progress in the computational capacity of mobile devices, there are many applications that are suitable to be executed using the MEC paradigm [1]. One of the most important scenarios in which the MEC paradigm can be used is data processing offloading, where applications are divided into small tasks that are executed in the edge nodes instead of being executed on the mobile device, either due to performance reasons or to save resources such as battery life [2]. Other MEC applications are mobile Big Data analysis [3], intelligent transportation [4] and service migration [5].

However, data processing offloading to a public cloud can lead to problems related to long waiting times during data transmission between the mobile device and the cloud. To solve this problem, the MEC paradigm was recently introduced. The main feature of MEC is to push network control, data storage and mobile computing to network edge nodes, closer to mobile devices, with a wide bandwidth connection and low round trip times, which allows for the execution of applications with real time needs [6]. MEC has introduced elements such as micro datacenters, cloudlets and fog that allow the development of multi-tier MEC architectures [7].

Security is one of the main challenges of the cloud paradigm, and it is a critical element of MEC implementation [8]. The infrastructure must guarantee important security aspects, such as confidentiality, authentication and integrity [9]. In addition, given that the security perimeter of edge computing tends to be weaker than that of a cloud computing, the edge computing must take into account security aspects inherent to the MEC paradigm [10].

Accordingly, studies on the security of MEC environments use, as the main security technique, one of the following methods: user authentication [7,11], application authentication [2], cloudlet authentication [12], data encryption [13], isolation techniques using a sandbox [14], data integrity [15] and trust mechanisms [16].

However, existing approaches lack certain characteristics that are present in the current MEC paradigm. On one hand, they do not consider new multi-tier MEC architectures and the security methods to be used in each of the tiers. In addition, they do not consider the different degrees of trust in each of the tiers; a private fog does not inspire the same trust as a public cloud or a cloudlet.

To address these deficiencies, this paper presents a security model for MEC scenarios that takes into account multi-tier MEC architectures and is based on the degree of trust of each tier. The basic idea is that different tiers have different degrees of trust associated with them, and therefore, they have different security requirements. A lower degree of trust requires a higher need for security, and vice versa. The key contributions of our work can be summarized as follows:

- The design of a security model for distributed mobile edge computing environments that is capable of adapting the security needs of each tier to its degree of trust.
- The proposed model can be applied to the security of modern multi-tier mobile edge computing architectures.
- The proposed model provides a formal framework for the externalization of application execution in multi-tier mobile edge computing environments. The general framework includes the aspects related to the security of these distributed architectures.
- An evaluation of the proposed model and architecture by deploying a three-tier mobile edge computing scenario and the running of a series of tests with different types of applications.

This study proposes a novel and flexible security model for data processing offloading that combines application to multi-tier MEC architectures with the ability to adjust the security level of each tier. This flexibility in terms of application to multi-tier MEC architectures is derived from the fact that each tier only needs local information of the subsequent tier. Furthermore, the system determines the required security level and appropriate security mechanisms, which means that the security level can be adjusted according to the degree of trust of each tier. Reducing the security level when the degree of trust is high improves performance.

The paper has been organized as follows: Section 2 presents a review of related studies; Section 3 describes the security model for mobile edge computing architectures, introduces the formal framework, and presents the system architecture while Section 4 evaluates the model by deploying a scenario and performs tests. Finally, the main conclusions of the research and future lines of work are presented.

## 2. Related Work

The scientific literature contains many articles that address the problem of cloud paradigm security [17,18]. Although most of these articles are focused on specific aspects of general cloud architectures, recently a growing number of contributions have dealt with specific security aspects related more explicitly to MEC models [19]. The following paragraphs analyze both general contributions to cloud security as well as specific contributions to MEC security.

In Ref. [9], a review of the state of the art of the cloud computing paradigm and the associated research challenges is presented. In the aspects related to security, the challenges mentioned include confidentiality and auditability. While confidentiality is addressed through encryption, the authors suggest that auditability should be provided at the hardware level—through reliable platform modules—and at the virtual level—through secure virtual machine monitors. Another study uses variable key sizes as a function of the confidentiality level of the data to improve reliability [20].

There is a proposal that is mainly based on two types of methods [21]. One method uses strong authentication mechanisms, based on technologies such as SSL (Secure Sockets Layer), digital certificates and Kerberos. The other method is the concept of data segregation, which refers to encrypting the users’ data storage to preserve the privacy of the users’ data.

Other proposals have addressed partial aspects of cloud security, such as an intrusion detection system for cloud computing environments offered as a service [22] or active monitoring schemes based on virtual machine introspection [23].

Given that this article is focused on the MEC paradigm, in addition to the previous proposals focused on the cloud paradigm, studies related to the security of MEC environments are reviewed.

User authentication is used as a security method to access the cloudlet using a Simple Authentication and Security Layer (SASL) in MEC environments [10]. In addition, the study points to future challenges such as the need to establish some type of trust between the user and the cloudlet. Another study about MEC that bases its security on user authentication techniques can be found in [2]. However, in contrast with the previous study, this study adds application authentication executed by the user through his/her mobile device. Finally, another system uses an authentication protocol for the mobile device to be able to authenticate the cloudlet [12].

The access control mechanism has been used in MEC systems. One system proposed a secure and verifiable outsourced access control scheme in fog-cloud computing [19]. In their scheme, most encryption and decryption computations are outsourced to fog devices and the computation results can be verified by using a multi-authority method. Another study describes a secure access control system that uses an intermediate layer between the mobile device and the cloud to control access; the intermediate layer is composed of cloudlets deployed by the cloud provider [7]. Additionally, a third system describes a framework for personal cloudlets that uses access control that is sensitive to user privacy [24]. Other approaches use proxy signatures as an access control protocol [25].

User privacy has also been analyzed in MEC environment [26]. The study presents a secure and privacy-preserving navigation scheme for fog-based vehicular ad hoc networks. Fog nodes cooperatively find the optimal route of a vehicle with conditional privacy preservation restrictions. Another study presents a platform that preserves privacy mainly through the use of two complimentary techniques: user authentication when using the cloudlet applications and isolation methods through the execution of applications in sandbox type mechanisms [14].

Finally, the trust of mobile users on fog or cloudlet providers has been taken into account in several recent studies. A system presents a security service recommendation system for fog users to share their own services, resources and data via efficient social networks [27]. Another study shows the need to establish measures to strengthen the trust in cloudlets [10], citing an example of the Trusted Platform Module (TPM) platform-based certification. Additionally, another system describes a trust management scheme for mobile cloudlet providers based on LTE mobile technology [16]. Finally, Marcon et al. [28] designs and develops a system for trust-based resource allocation on large-scale cloudlet platforms.

As described in previous paragraphs, there are several studies related to the security of MEC environments. The existing proposals make use of a wide range of security methods: user authentication, application or fog/cloudlet authentication, isolation or sandbox methods and trust schemes. However, existing proposals do not consider the new multi-tier architectures of the MEC paradigm [1]. In addition, they do not account for the different degrees of trust that each of these tiers or each fog/cloudlet provider may have.

This study presents the design of an MEC security model based on the degree of trust of the different tiers that intervene in the MEC paradigm. The main idea is that the tiers that intervene in any MEC deployment have different degrees of trust and therefore require different security levels. For example, generally speaking, the degree of trust will be higher in a private fog than in a public fog. However, the degree of trust will be higher in a public cloud than in a public cloudlet because the cloud generally belongs to large organizations with great resources to provide security and auditing mechanisms of their systems, while a cloudlet may be provided by domestic users with fewer resources and may be next to impossible to audit [16].

In addition, the security model can be applied in multi-tier MEC architectures because each tier must only fulfill the security requirements of the next tier with which it communicates, and these requirements will depend on the degree of trust placed in the subsequent tier. For example, a mobile device will be able to communicate with a private fog maintaining the security requirements of the fog, and in turn, the fog will be able to establish communication with a public cloudlet by fulfilling the security requirements of the latter. Information is needed to fulfill the requirements of the next tier, for example, if the next tier requires authentication, it will be necessary to have the correct credentials.

## 3. Secure Multi-Tier Mobile Edge Computing Model

We will now analyze the main aspects of the security model designed for the MEC computing paradigm. First, we will define the formal framework of the model in which multiple tier MEC computing is developed, highlighting the formalism related to the security of these types of systems [29]. Then, we will focus on the study of a use case of the security model for the proposed MEC paradigm. Finally, we formulate a system architecture that allows for application to real conditions.

### 3.1. Formal Framework

This section introduces a general formulation of the multi-tier MEC paradigm and, more specifically, the aspects related to data processing offloading architectures. In addition, because security is the main purpose of this study, we go in-depth and focus on the formulation related to the security of MEC models in computational offloading scenarios.

First, it is necessary to consider that an application will be composed of a list of tasks to perform. There may be dependent tasks that need sequential execution and independent tasks that can be executed in parallel in different tiers. However, splitting an application into a list of tasks is a problem called application partitioning that is outside the scope of this paper. We will generally make use of the concept of task with independence from its granularity unit, which may be of variable size. The granularity unit can be a fragment of code, a method, class, process, etc.

Let Γ_App_ be the list of tasks of an application:

Γ_App_ = {t_1_, t_2_, …, t_n_} (1)

Because we are formally modeling multi-tier MEC architectures in data processing offloading scenarios, the tasks of an application can be executed in any of the MEC tiers or layers. In the rest of the paper, we will use tier or layer indistinctly. Let ℒ be the set of available computing layers of the MEC architecture:

ℒ = {L_0_, …, L_c_} (2)

The MEC architecture may have any number of layers between 2 and c + 1. L_0_ is the layer made up by the mobile device in which the application initiates and, if c + 1 layers are used, Lc is the layer in which the remote Cloud Computing servers are located. The number of layers of the MEC architecture will depend on the execution environment, and different environments can have a different number of layers. For example, a user in his home environment may have only two layers: the mobile device (L_0_) and the fog servers of his home (L_1_). But, the same user in his work environment may have three layers: the mobile device (L_0_), the company’s fog servers (L_1_) and the company’s ISP cloud servers (L_2_).

In general, each of the layers of a multi-tier MEC architecture can have a set of heterogeneous computation platforms with different processing and performance capacities. Thus, the layer L_j_, has m computing platforms where j ∈ {0,c} and m > 0.

L_j_ = {p_j1_, p_j2_, …, p_jm_} (3)

where p_jk_ is the platform k of the layer j. In general, the number of platforms (m) will be different in each layer, for example, a private cloudlet usually has several servers while a large public cloud has thousands of servers.

At any given time, for a mobile device, there is a list of platforms that belong to each of the upper layers to offload the code. In other words, a device can offload the tasks to a specific fog node, cloudlet server, base station or cloud server, but normally, it cannot decide which of the platforms of a layer to use. This depends on several aspects such as geographical location, etc.

For a specific mobile device i, the list of higher MEC layer platforms available can be expressed as A_i_:

A_i_ = {p_1,i_, p_2,i_, …, p_c,i_} (4)

The set of platforms described establishes the specific multi-tier MEC architecture of a mobile device and expresses the data processing offloading possibilities of the device applications at a given time. The list of platforms of a mobile device depends on the environment and may vary with time as a function of different situations. For example, it may vary based on changes in the availability of infrastructure or changes in the location of the mobile device (home, office, etc.).

There are currently multiple mechanisms or methods to provide security to computational systems. Some of the most widely used methods are user authentication using different types of credentials [30], server authentication mainly using digital certificates [12], confidentiality through the use of encryption mechanisms [31], firewall systems for filtering information [32], intrusion detection systems [33,34], integrity using digital signatures [35], application and data isolation [16], etc. Let M be the set of available security methods:

M = {M_1_, …, M_p_} (5)

The previous methods allow for adding security to the computer systems independent of their architecture and heterogeneity. In addition, each of the security techniques can be used individually or in combination with others. As the number of security methods used increases and as long as they are combined adequately, the system security is improved. Therefore, different security levels can be established, with S being the set of possible different security levels:

S = {S_1_, …, S_q_} (6)

The order of the different levels is important, S_1_ is the least safe level and S_q_ is the safest level. The cardinality of S is not determined, in general, few security levels are usually defined. For example, Microsoft Forefront has five security levels.

M and S are related by the fact that a higher level of security is associated with the use of more and better security methods. This relationship is expressed in the definition (9). We can extend the formalism of the framework to include different degrees of trust that each of the layers of a distributed multi-tier MEC architecture may have. Therefore, let T be the set of different degrees of trust that each of the different layers of a distributed multi-tier MEC architecture may have:

T = {T_1_, …, T_q_} (7)

Of course, logic dictates that there is an inverse relationship between the degree of trust of a layer and its security needs. The basic idea is that the higher the degree of trust in a layer of the model, the lower the security level that will be required to establish communication with this layer. In contrast, if the degree of trust is lower, a higher level of security will be required.

Taking into account this key concept, we can establish a relationship between the degree of trust of any layer of the distributed multi-tier architecture and the level of security that this layer requires. In other words, we can determine the level of security of a model layer based on the degree of trust placed in this layer. Therefore, the following expression defines § as the function that returns a specific level of security for a given degree of trust:

§(T_j_) = S_i_ (8)

where T_j_ ∈ T and S_i_ ∈ S. Since the cardinality of T and S is the same (q), there is a direct relationship between a specific degree of trust and its corresponding level of security. After determining the different degrees of trust and different security levels, we must establish the set of security methods that will be applied in each of the levels. In this case, a higher security level requires a higher number of mechanisms to guarantee the level’s security. We can establish the function Φ as a function that yields the list of security methods that we must use for a given security level:

Φ(S_i_) ≡ ‹M_1_, …, M_k_› (9)

where S_i_ ∈ S, M_1_ ∈ M and M_k_ ∈ M.

In this case, the order of security methods is not important, therefore ‹M_1_, …, M_k_› = ‹M_k_, …, M_1_›. What is really important is to use all the methods determined by the function Φ, because the level of security is guaranteed only if all the methods are used.

After establishing the different sets of elements present, it is necessary to be able to determine the behavior of any computing paradigm and, in this specific case, the behavior of the secure MEC models with multi-tier architecture.

To determine the behavior, we will analyze the system performance and we will measure this performance as cost of execution (E) in terms of time. The cost of execution of an application in the distributed multi-tier MEC infrastructure is determined by three main components, as described in expression (10): (a) the cost of computation (Cmp); (b) the cost of communication throughout the network and its tiers (Net); and (c) the cost of security (Sec):

E(Γ_App_) = Cmp(Γ_App_) + Net(Γ_App_) + Sec(Γ_App_) (10)

In expression (10), we focus on the component related to security, because this paper addresses the security of the multi-tier MEC paradigm. Therefore, the objective is to independently determine the cost associated exclusively with the security of these distributed architectures.

The cost of execution depends on the environment’s dynamics, i.e., the execution conditions change based on aspects related to the work load of each platform and the amount of network traffic. The optimal placement of edge servers in MEC environments is able to optimize the mobile edge computing network performance [36].

In expression (10), the computation cost component is related to the processing capacity of the different computing platforms in each of the layers of the distributed MEC architecture. Of course, the higher the processing capacity of the platform is, the lower the associated computing cost associated with it. We can determine the computation cost of a task t_i_ of an application in any platform of the MEC architecture. The following expression shows the computation cost of a task t_i_ in platform j of layer k of a multi-layer architecture. The cost will be a positive real value:

Cmp(t_i_, p_k,j_) ∈ ℝ+ ⋃ {0} (11)

where ℝ+ ⋃ {0} means the set of positive real numbers, including zero.

Similarly, the cost of execution in expression (10) also depends on the cost of communication throughout the different layers of the architecture. This second component in turn depends on the bandwidth and dynamic aspects such as the network traffic present in these links, which may generate bottlenecks or even disconnections at specific points or failure of certain links.

Based on this, the cost of communication of moving tasks and their associated data between platforms in different layers can vary according to the state conditions and workload of the communication network. Expression (12) describes the cost of communication of moving a task t_i_ from the mobile device to platform j in layer k:

Net (t_i_, p_k,j_) ∈ ℝ+ ⋃ {0} (12)

Finally, the cost of security is related to both the processing capacity of the platforms as well as the quality of the links in the networks that connect the MEC architecture tiers. For example, encrypting communications to guarantee the confidentiality of the information will be related to the processing velocity of the corresponding encryption algorithms and therefore to the computation capacity of the platforms. On the other hand, implementing authentication mechanisms will be related to the communication velocity of the credentials of the layers involved. The following expression shows the cost of security associated with the execution of a task t_i_ in platform j of layer k of a multi-tier architecture:

Sec (t_i_, p_k,j_) ∈ ℝ+ ⋃ {0} (13)

By analyzing these three cost components and setting aside the dynamic environmental conditions (workload, network traffic, etc.), we observe that the computation cost and the cost of communication are fundamentally related to only one principal variable. The cost of computation is related to the processing capacity of the platform, and the cost of communication is related to the quality of the communications link, which is directly related to connection bandwidth.

However, the cost of security depends on multiple variables determined in the design phases and during configuration of the computational systems; in other words, the cost of the security will be directly related to the number of security mechanisms or methods used. Of course, the more security mechanisms that are used the more the total cost of security is increased.

Each of the security methods has an associated cost that is independent of the cost of the other methods. There are methods related to the processing and communication capacity, for example, authentication methods that depend on transmitting user or server credentials and subsequent verification in the database. On the other hand, there are methods that fundamentally depend on the processing capacity, for example, encryption done to guarantee the confidentiality or verification of digital signatures to guarantee integrity.

Based on this information, we will define the set of CM as the set of security costs of each of the existing methods defined in Equation (5):

CM = {CM_1_, …, CM_p_} (14)

The different MEC architecture layers can incorporate different numbers of security methods based on their own requirements. In other words, the distributed architecture layers can have different security levels with a combination of methods for inherent security and therefore different costs. In addition, the costs will depend on the capacities of the platforms in the layers. The top layers of multi-tier MEC architectures are those that provide services to the lower layers because they have higher computation and storage capacities. In general, the higher the number of services offered, the higher the level of security required. Given that each security level has its own cost, let CS be the set of security costs of each of the security levels defined in Equation (6).

CS = {CS_1_, …, CS_q_} (15)

Taking into account the previous sets and equations, we can determine the cost of security of an application based on the list of layers and platforms in which each task is executed.

We can define ∧_i[k,j]_ = (t_i_, p_k,j_) to represent that the task t_i_ is processed on platform j of layer k. Then, the security cost of executing task t_i_ on platform j of layer k is defined as:

Sec(∧_i[k,j]_) = CS_k_ = ∑_l_ CM_l_ (16)

Sec(∧_i[k,j]_) will be equal to the security cost of the layer k where the task t_i_ is executed, which will depend on the level of security of the layer. Finally, Sec(∧_i[k,j]_) will be the sum of the security costs of each security method used in that layer of the MEC architecture.

Based on the above definitions, the sequence of layers and platforms on which the application runs is defined by the vector ∧(Γ_App_) as follows:

∧(Γ_App_) ≡ ‹∧_1[k1,j1]_, …, ∧_n[kn,jn]_› (17)

In the above definition, ∧_1[k1,j1]_ represents the execution of task t_1_ on platform j_1_ of layer k_1_ and ∧_n[kn,jn]_ indicates execution of task t_n_ on platform j_n_ of layer k_n_.

Finally, the execution cost of an application can be obtained by expanding expression (10) with the layers and platforms of the vector ∧:

E(Γ_App_) = ∑_i_ [Cmp(∧_i[k,j]_) + Net(∧_i[k,j]_) + Sec(∧_i[k,j]_)] (18)

### 3.2. Use Case

As defined in the general framework of the model, it is possible to establish as many degrees of trust and security levels as one desires as well as to use any number of available security methods. However, for the sake of simplicity and clarity, this section proposes a use case with three degrees of trust, three security levels and five security methods. The following expressions describe the sets of degrees of trust, levels and methods that are used in the use case:

T = {T_1_, T_2_, T_3_} (19)

S = {S_1_, S_2_, S_3_} (20)

M = {M_1_, M_2_, M_3_, M_4_, M_5_} (21)

As commented before, according to the general framework, we can have as many degrees of trust in the layers of the distributed architecture as we want to have. However, this use case will only establish three degrees of trust, as follows:

- *High trust*—corresponds to those layers of the MEC architecture in which there is total trust. In general, the layers of high trust will belong to the administrative domain itself, i.e., private fog, cloudlets or clouds which there is administrative authority. In addition, this degree of trust may also be assigned to public providers with SLA contracts for which there is certainty that they have significant security measures and third-party auditing systems [16].
- *Medium trust*—corresponds to those MEC layers in which there is some trust but over which there is no type of administrative authority. This degree of trust corresponds to fog or cloudlets offered by a provider of services with a measure of reliability and with which there is an SLA contract. A characteristic that defines the medium degree of trust is the limited security and auditing resources. This degree of trust will also be assigned to public providers with SLA contracts for which there is no certainty regarding the implementation of strong security measures and, especially, to those that do not have independent auditing systems.
- *Low trust*—corresponds to those MEC layers for which there is no trust. There will be no administrative authority, and there will also be no service contract associated with them. The low degree of trust will be assigned to public providers without a service contract and which can independently decide on security and auditing measures they implement.

After defining the different degrees of trust, we will establish the level of security of each of the three degrees of trust. Like the degrees of trust, the set of security levels established in the general framework can have as many levels as required. This use case will only define three security levels that will be related to the three degrees of trust. However, a different number of degrees of trust or security levels could have been established.

As previously indicated, a higher degree of trust requires a lower level of security, i.e., there is an inverse relationship between the degree of trust of a layer in the model and its security level. This relationship will be determined by the function introduced in expression (8), which is based on degree of trust and yields of associated level of security. In this use case, we have manually set the three security levels, but they could be calculated automatically by defining a specific function according to the expression (8). In our case, the three security levels will be as follows:

- *Security level S*_1_*.* When a layer i of the MEC architecture is related to another layer i+1 with which there is a high degree of trust, an S_1_ security level will be established. The security level S_1_ will require the implementation of the fewest security measures.
- *Security level S*_2_. This will be the security level established for those layers of the distributed architecture over which the degree of trust is medium. This level of security will require more security measures than the previous level.
- *Security level S*_3_. The security level S_3_ will be defined for those layers of the architecture that have a low degree of trust. Of course, this level will incorporate the highest number of security measures.

In the rest of the paper the word element will refer to any computing platform, for example, a mobile device, a fog node, a cloudlet server, a cloud server, etc. When an element of the multi-tier MEC architecture establishes a relationship with another element of a higher layer in which there is a high degree of trust and, therefore, the security level is determined to be low, it will be necessary to use the following security methods:

- *User authentication.* The element corresponding to the higher layer of the multi-tier architecture will carry out the authentication of the user that is establishing the communication. For example, if a mobile device establishes a connection with a private fog node, it will be necessary for the private fog node to carry out the user authentication of the mobile device.
- *Fog/cloudlet authentication.* The element of the lower layer of the distributed architecture will carry out the server authentication of the higher layer with which communication is being established. For example, in the previous case, the mobile device must use the private fog node certificate to guarantee the authenticity of the fog node.
- *Encrypted communications.* Although the degree of trust may be high, for security reasons, the exchange of information must always be encrypted using technologies such as SSL/TLS.

In summary, for a high degree of trust, the elements must use communication encryption and double authentication in both directions of the communication. On the one hand, user authentication is needed for the MEC element to validate the user that is connecting from his/her mobile device. On the other hand, fog/cloudlet authentication is needed for the device to authenticate the fog or cloudlet with which it will establish a communication.

If the degree of trust between the elements that intervene in the communication is medium, then the level of security will also be medium (S_2_), and therefore, the security needs will be greater than in the previous case. Specifically, when the security level is S_2_, the three security methods of the previous level will be used: user authentication, fog/cloudlet authentication and connection encryption. However, in addition to the three previous methods, isolation techniques or sandboxing will also be used on both sides of the connection so that the information on both MEC elements is completely isolated from other applications and the communication can only occur between the two sandboxes involved.

Finally, a low degree of trust will demand the highest level of security, which is why more security techniques will be needed than for the medium degree. Specifically, we have added integrity management through digital signatures to the methods used in the security level S_2_. By using this security method, we periodically verify the digital signature of the tasks that have been taken to the higher layers of the distributed MEC architecture for execution to guarantee their integrity.

**Figure 1:**
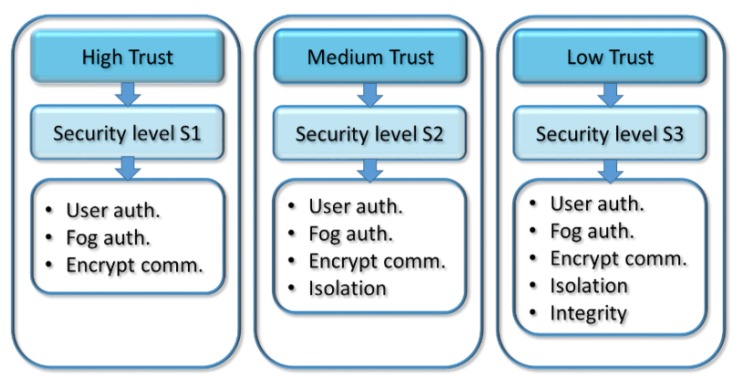
Use case of the secure MEC model based on the degree of trust.

Figure 1 shows the use case of the security model with different degrees of trust and security levels defined, as well as the security measures required in each of the levels. Although this use case has defined three degrees of trust, three security levels and five security methods, it is important to emphasize that we can establish as many degrees of trust and security levels as we want, and for each of them, we can use many different combinations of security methods. The key idea of the proposal is that we can adapt the level and methods of security used in relation to the degree of trust of the elements in the MEC architecture, independently of the degree of trust, levels and methods used.

The algorithms or security methods used are the following: user authentication by username and password; fog/cloudlet servers authentication using self-signed X509 certificates with openssl; communications have been made using https connections using openssl for encryption; isolation via Android Application Sandbox and servlets; and, digital signatures based on the SHA-256 algorithm were used to verify the integrity of downloaded applications. 

**Figure 2:**
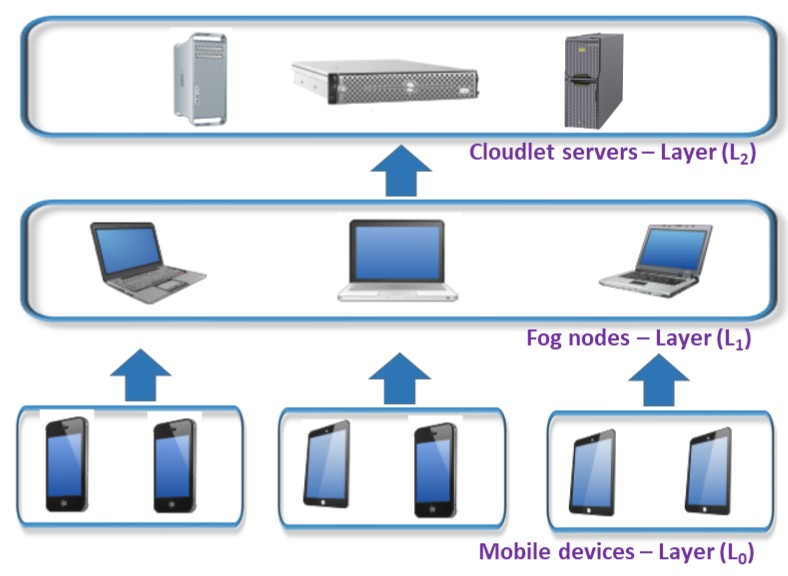
Classical three-tier MEC architecture.

After defining the use case of the security model based on the degree of trust of the different layers of the MEC architecture and establishing the different security needs as a function of the degree of trust, we will show that the model can be applied to multi-tier MEC architectures. Specifically, we will apply the use case to a classical three-tier MEC architecture: mobile devices, fog nodes and cloudlet servers. This classical architecture is shown in Figure 2.

Let us suppose that the fog nodes of the intermediate layer (L_1_) belongs to a service provider with which we have an SLA contract and that the cloudlet has no contract (L_2_). According to our previous description, the degree of trust placed in the fog will be medium and the degree of trust placed in the cloudlet will be low. Therefore, to establish communication between the mobile devices (layer L_0_) and the fog nodes (layer L_1_), it will be necessary to have a double authentication process; the fog will authenticate the user of the mobile device, and the mobile device will authenticate the fog node. In addition to the double authentication, the communications will be encrypted as well as isolated for both the mobile device and the fog node. In addition, for the intermediate level fog node (L_1_) to communicate with the cloudlet server (L_2_), it will be necessary to have not only the double authentication of the previous level, encrypted communications and application isolation but also the integrity of the applications guaranteed using digital signatures when offloading to the cloudlet server. As shown, the model can be extended to multi-tier MEC architectures in which each layer establishes the security measures only for the layer with which it needs to communicate, which in turn will depend on the degree of trust placed in the subsequent layer.

### 3.3. System Architecture

**Figure 3:**
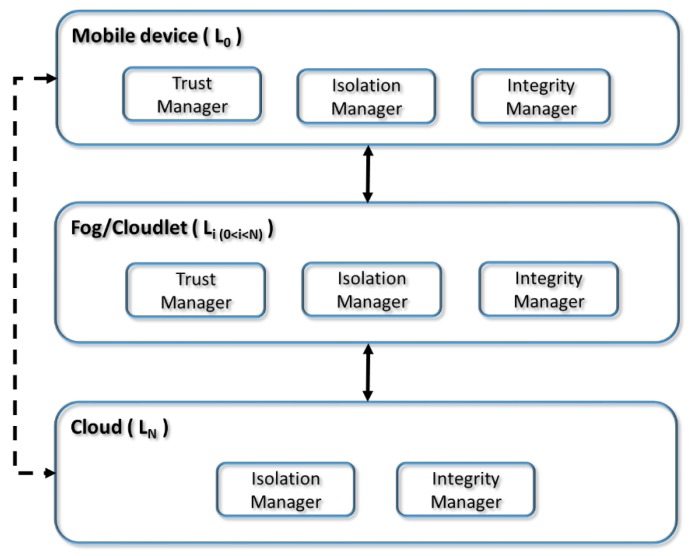
System architecture for a multi-tier MEC paradigm deployment.

In this section, we will describe the system’s architecture by determining the different components derived from the proposed use case as well as their distribution within a multi-tier MEC architecture. Figure 3 shows the components of the architecture and their distribution in the different MEC layers.

We have designed an architecture with three modules or principal components: trust manager, isolation manager and integrity manager. The security methods user authentication, fog/cloudlet authentication and communications encryption are not included as specific modules of the architecture because they are already well established elements that are present in all systems. These methods use user credentials and digital certificates, which will be transmitted through SSL/TLS tunnels.

When a user wishes to establish a connection through his/her mobile device with a fog or cloudlet, the first thing the user must do is to determine the degree of trust of the fog or cloudlet. The trust manager module is in charge of determining the degree of trust of the layer with which connections are to be established. There are different approximations to carry out this function, ranging from protocols—that establish trust through verifications before use—to trust schemes—based on reputation systems [16]. In our use case, we have manually defined the degree of trust in based on compliance with a set of conditions, which include belonging to the same administrative domain, having an established SLA contract, etc.

In addition, when one of the layers that intervene in the connection has a medium degree of trust, it is necessary to use isolation techniques on both ends of the communication. The isolation manager module will be responsible for implementing the different techniques that allow for the highest possible level of isolation on both ends of the connection. To achieve this, techniques such as virtual machines and containers that are conveniently fortified for processing and storage will be used. In addition, perimeter security techniques will be used for communication isolation.

Finally, in the case of the layers that have a low degree of trust and demand a high security level, in addition to all the methods used in the medium level, it is necessary to guarantee the integrity of the tasks and applications. The integrity manager module will be in charge of validating the integrity of the applications that are offloaded from the mobile device to the low trust layer through the use of digital signature methods. To that end, the integrity manager will periodically verify the digital signature of the applications or tasks that are executed in the fog or cloudlet through data processing offloading.

The distribution of the three principal modules of the architecture in the different MEC layers is not uniform, as can be seen in Figure 3, which shows the architecture of the system for a three-tier MEC paradigm deployment (mobile device, fog and cloudlet). The reason is that each of the layers of a multi-tier MEC architecture has different security needs or functionalities.

The mobile device (layer 0) and the intermediate layers of the MEC architecture, typically fog and cloudlets, do require the three modules: the trust manager—to obtain the degree of trust of the next layer with which it communicates—; the isolation manager—to isolate tasks and communications—; and, the integrity manager—to periodically check the integrity of the tasks—. The last layer of the MEC architecture, generally corresponding to public clouds, which can only be servers, do not have any subsequent level and do not need the trust manager module.

Note that the security model’s architecture allows the mobile device to connect only with one fog or cloudlet (continuous line) or only one cloud (dashed line), which is a two-tier MEC architecture. However, it also allows the connection to the cloud through k fog or cloudlet layers (continuous lines), establishing a multi-tier architecture. In addition, given that the intermediate MEC layers have all the modules of the security use case, it is possible to have multiple intermediate layers. Therefore, the security model and its architecture can be applied in multi-tier MEC architectures. 

In summary, the multi-tier security platform, for this use case, consists of three main functional blocks: trust manager; isolation manager; and, integrity manager. These three components are necessary because they perform specific functions that are not usually present by default in existing systems. All tiers need all three components except for the last tier (L_N_), which does not need the trust manager. It is important to consider that there is no direct relationship between the different functional blocks. Furthermore, other use cases may need or define different functional blocks, for example, two-factor authentication. In addition, the model uses user/fog/cloudlet authentication and encryption are not shown in the architecture because they are well-known functional blocks and are present in all systems.

## 4. Results

To perform the necessary tests to validate the proposed model, a prototype has been created with all the components of the architecture. To implement it, we have used the Android 5.1 (Lollipop) platform in the mobile device and Apache Tomcat 8 as a container of Java servlets on the fog/cloudlet side. The trusted computing base (TCB) consists of the software mentioned, the Linux operating system used in the fog/cloudlet server and the different implemented components of the security model. 

The tests focus on the main goal of the proposal, which is to evaluate the security platform’s performance. However, the tests do not deal with the effects of communication bandwidth and processing capacity, which would require another type of test.

User authentication has been implemented through user and password pairs stored in a database. The authentication of fog/cloudlet servers was done through certificates generated manually without signature by a certifying authority. To verify the integrity, we have used digital signatures based on the SHA-256 algorithm.

Isolation in the mobile device rests on the Android Application Sandbox based on the user protection and isolation mechanisms of the Linux core. The Android Sandbox assigns a unique user identifier to each application and executes it in a separate process. Therefore, the Android platform reinforces security by providing isolation by processes and users, while other operating systems only isolate processes and allow multiple applications to execute with the same user permits.

Isolation on the fog/cloudlet server end has been implemented at the application level and at the level of the communication channels. The applications have been implemented through servlets that are executed while isolated in their own ServletContext, and Tomcat has been configured to prevent information from being passed between contexts (property crossContext = “false”). In addition, to avoid a servlet from addressing requests from several users, which risks sharing confidential data, multiple instances of the servlet are created so that each user is attended by a different instance in a different context. In terms of isolating the channels of communication, the mod_security module by Apache has been used to insert traffic rules in the firewall and allow or isolate only the communication between the IP address and user remote ports and between the IP address and the servlet port in the fog/cloudlet server.

We have performed tests using Energy Phone Max 4000 mobile devices (Quad-core at 1.3 GHz, Android 5.1 and 3G connectivity, Energy Sistem Technology S.A., Alicante, Spain) in layer 0 (mobile device), computers with Intel Core i7-4790 CPUs (Intel Corporation, Santa Clara, CA, USA) with four cores and 16 GB RAM in layer 1 (fog nodes), and servers with Intel ® Xeon® E5-2630 v4 processors (Intel Corporation, Santa Clara, CA, USA) with 10 cores and 32 GB of RAM memory in layer 2 (cloudlet servers). The operating system executed on the PCs was Linux Ubuntu 16.

The purpose of the first test is to measure the overhead of the different security mechanisms used in the model with respect to a base scenario without security. Given that one of the principal scenarios of application for the model is the data processing offloading from a mobile device to a fog/cloudlet when the application to be executed is computationally intensive, the workload consisted of transferring data blocks of different sizes, in multiples of 100 KB, calculating the data block hash through the SHA-256 algorithm and returning the results. The connection between the mobile device and the fog/cloudlet node was achieved using Wireless LAN [37].

**Figure 4:**
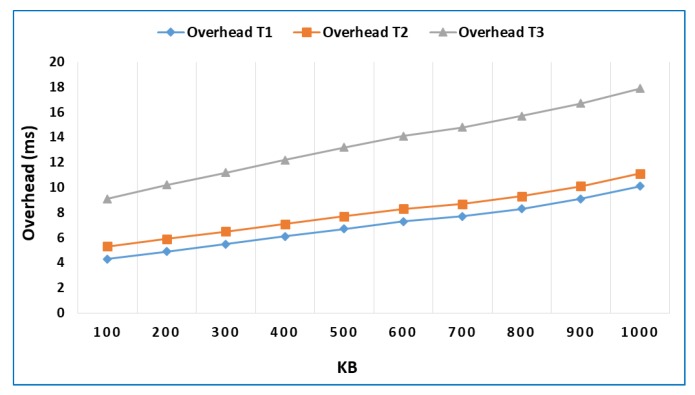
Overhead for each security level.

Figure 4 shows the comparative results of the performance of security methods used in each of the different degrees of trust, with respect to the base scenario in which no security method was implemented.

It can be seen in Figure 4 that a high degree of trust generates the lowest overhead of the three security levels analyzed. In the worst-case scenario, it adds 6 ms of overhead in terms of execution time, with respect to not using any security method. It can also be seen that the medium degree of trust adds a very small overhead when compared to the reference case. In other words, adding isolation techniques to the execution of applications barely has a cost. This is because existing technologies have been used in the platform and they only have to add a pair of rules to the mod_security firewall application to isolate the channels of communication between applications. Lastly, the low degree of trust, which includes integrity management as an additional security method, adds an overhead that is very similar to the high degree of trust case, with the total overhead being approximately twice the overhead of the first case.

**Figure 5:**
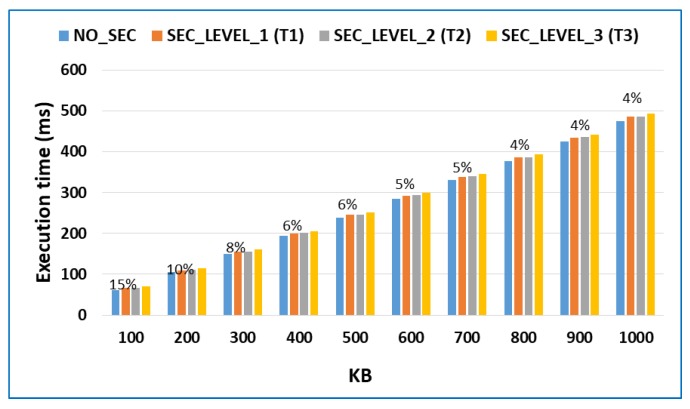
Overhead percentage in intensive applications.

Figure 4 measures the overhead of the different security levels of the model using an absolute value and shows that a higher security level results in more methods being used, which in turn results in a higher overhead. However, it is convenient to consider the overhead as a percentage of the increase in execution time with respect to the execution without security. Figure 5 shows this point of view.

Figure 5 shows that the overhead percentage of the security model decreases as the computation and communication needs of the application being executed increase. Specifically, with a low degree of trust and high security level, the overhead level is high (15%) for applications with low communication cost (Net) and computation cost (Cmp). In contrast, the overhead is low to medium as the execution cost of the application increases (4%). In addition, it tends to stabilize at a low security cost (Sec), as the other components of the execution cost go up.

The purpose of the previous test was to analyze an application with both computation and communication costs. However, the type of application that is best suited to data processing offloading is an application that mainly has a high computational cost [38]. For this reason, the following test intends to measure the overhead of the security model in these types of applications. The test consists of calculating all prime numbers from 1 to 1000, and the calculation is repeated a specific number of times, from 100 to 1000.

**Figure 6:**
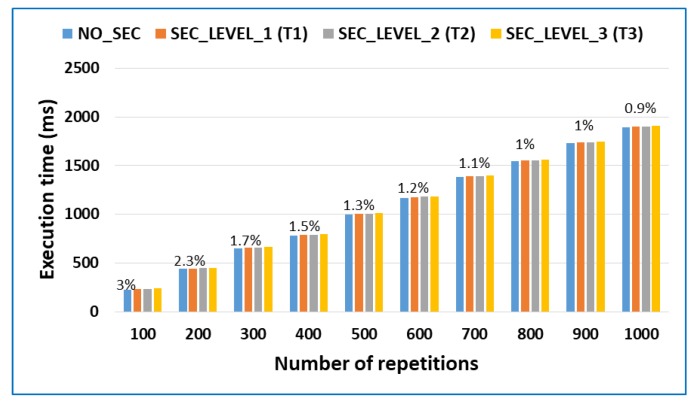
Overhead percentage in applications that are intensive only in terms of computational cost.

Figure 6 shows the results of the test. As can be easily verified, the overhead percentage of the security model in applications based on computational cost is very low. Specifically, with the maximum security level, the computation cost exceeds 3% when the calculation is repeated 100 times, but it is approximately 1% for the majority of the cases. Therefore, the security model produces a slight overhead on the type of applications that best utilize external execution for mobile device using data processing offloading techniques. We must also keep in mind that the higher the computational cost of the application, the lower the overhead of the security cost will be because certain security methods are only applied once (user authentication, cloud authentication, isolation) and are independent of the computational workload.

In addition, executing the same application in the mobile device resulted in execution times that varied from 604 ms for 100 repetitions to 5832 s for 1000 repetitions. Therefore, executing the application in a secure way on the fog node is much faster than executing it on the mobile device.

**Figure 7:**
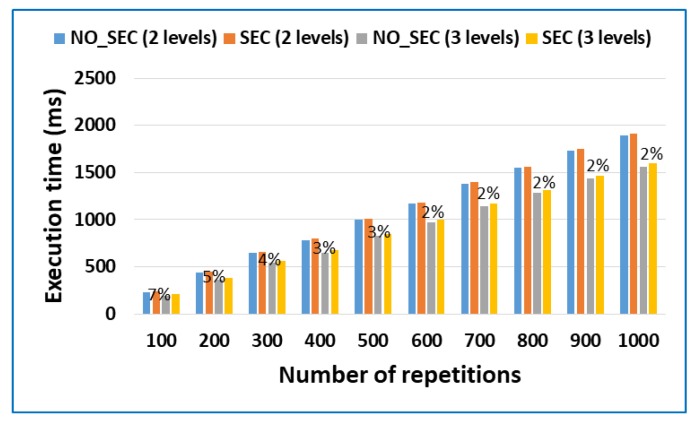
Overhead percentage in three-tier MEC architectures.

Additionally, a test was performed to show that the security model can be applied in multi-tier MEC architectures. To perform the test, we used three tiers: the mobile device, the fog tier with the Intel Core i7-4790 computers and the cloudlet with the Intel^®^ Xeon® E5-2630 v4 servers. The test considered the fog and cloudlet to be a low trust connection, and therefore, only the maximum security level was used, both between the mobile device and the fog and between the fog and the cloudlet. The test consisted of calculating the prime numbers, like the previous case. In the case of three tiers, the calculation has been divided between the fog and the cloudlet by 50%. Figure 7 shows the results.

Figure 7 shows that the model can be applied to three-tier MEC architectures. In addition, this proves that the model can be extended to multiple layers because in the model, layer L_i_ only takes into account the degree of trust of layer L_i+1_ to determine the security mechanisms to be used, independently of the value of i.

The results show that for the specific case of this application, execution of the application in a three-tier architecture produced the best results in terms of performance and execution time. Specifically, we obtained an improvement of approximately 20%, taking into account that only 50% of the application was executed in the cloudlet.

In addition, we can see that the overhead percentage of the security model in the three-tier architecture, in the majority of cases, is between 2% and 3%. The percentage is higher than that obtained in the case with two layers because the majority of the security methods have to be performed twice for architectures with three layers.

Finally, following an approach similar to [39,40], We have carried out a test to check the security of the proposed model. The test launches attacks against applications that are running on fog servers: local applications and mobile device applications with the proposed security system. The test tries to determine if the security system reduces the impact of attacks on applications.

**Figure 8:**
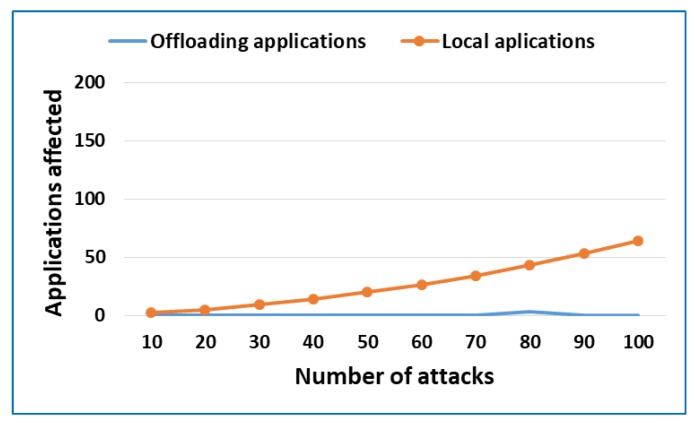
Security of the proposed model against common attacks.

The scenario consisted of a set of fog nodes executing 200 local applications and several mobile devices that run their applications on these nodes through data processing offloading. While the applications are running, the fog nodes have been subjected to a set of attacks obtained from public sources such as exploit-db.com, attacks include local and remote exploits such as Tomcat JSP upload bypass or JMX code execution. Figure 8 shows the results of the tests.

The results show that as the number of attacks increases the local applications affected by the attacks increases considerably. Specifically, when 100 attacks are launched, 64 local applications are affected. However, mobile applications executed on the fog nodes through data processing offloading are not affected. Only 3 mobile applications have been affected when 80 attacks have been launched. The good results obtained are due to the security measures implemented as well as the fact that mobile applications that run on fog nodes are only a small period of time, reducing the opportunity for attacks.

## 5. Conclusions

This study presents a security model for mobile edge computing architectures that has the capacity to adapt the level of security of the different elements in the architecture to the degree of trust of each of those elements. The model determines the number of security mechanisms to be used in each element as a function of the degree of trust of that element. The key idea is that a higher degree of trust between the parties requires a lower security level, and a lower degree of trust requires a higher security level. Of course, the higher the security level required, the higher the number of security methods used.

In addition, the model can be applied to modern mobile edge computing architectures with multiple tiers or layers because each layer will determine the degree of trust with another layer and will only have to adapt the security level to that degree of trust. As a result, the model establishes a chain of trust from the mobile device (edge) to the public cloud (core), where each layer establishes the necessary security measures associated to the degree of trust of the following layer.

A formal framework was introduced, which allows for the expression or representation of data processing offloading from the mobile device to different tiers of a multi-tier mobile edge computing architecture. In addition, we have included in this general framework the aspects related to the security of the MEC paradigm. An architecture was developed that allowed the implementation of the model in real environments. Finally, tests demonstrated that the model only imposes a minimum overhead in terms of execution cost, which means it can be applied in real environments.

Finally, this study lays the foundation for a series of future lines of research. Further research to automatically determine the degree of trust of the distributed architecture elements is necessary. A starting point is trust management systems based on reputation mechanisms. In addition, it would be convenient to study in-depth the combination of security mechanisms that must be established to guarantee each security level.

## Fundings

This work was supported in part by the Conselleria d’Educació, Cultura i Esport, Generalitat Valenciana. Grant GV/2015/122.
